# Structural insights into the γ-lactamase activity and substrate enantioselectivity of an isochorismatase-like hydrolase from *Microbacterium hydrocarbonoxydans*

**DOI:** 10.1038/srep44542

**Published:** 2017-03-15

**Authors:** Shuaihua Gao, Yu Zhou, Weiwei Zhang, Wenhe Wang, You Yu, Yajuan Mu, Hao Wang, Xinqi Gong, Guojun Zheng, Yue Feng

**Affiliations:** 1 Beijing Key Lab of Bioprocess, the State Key Laboratory of Chemical Resources Engineering, College of Life Science and Technology, Beijing University of Chemical Technology, Beijing 100029, PR China; 2 National Institute of Biological Sciences, Beijing, No. 7 Science Park Road, Zhongguancun Life Science Park, Beijing 102206, PR China; 3 Key Laboratory for Protein Sciences of Ministry of Education, Tsinghua-Peking Center for Life Sciences, School of Life Sciences, Tsinghua University, 100084, Beijing, PR China; 4 Institute for Mathematical Sciences, Renmin University of China, Beijing 100872, PR China

## Abstract

(+)-γ-lactamase catalyzes the specific hydrolysis of (+)-γ-lactam out of the racemic γ-lactam (2-Azabicyclo[2.2.1]hept-5-en-3-one) to leave optically pure (−)-γ-lactam, which is the key building block of antiviral drugs such as carbovir and abacavir. However, no structural data has been reported on how the enzymes bind the γ-lactams and achieve their enantioselectivities. We previously identified an isochorismatase-like hydrolase (IHL, Mh33H4-5540) with (+)-γ-lactamase activity, which constitutes a novel family of γ-lactamase. Here, we first discovered that this enzyme actually hydrolyzed both (+)- and (−)-γ-lactam, but with apparently different specificities. We determined the crystal structures of the apo-form, (+)-γ-lactam bound, and (−)-γ-lactam bound forms of the enzyme. The structures showed that the binding sites of both (+) and (−)-γ-lactam resemble those of IHLs, but the “cover” loop conserved in IHLs is lacking in the enzyme, probably resulting in its incomplete enantioselectivity. Structural, biochemical, and molecular dynamics simulation studies demonstrated that the steric clash caused by the binding-site residues, especially the side-chain of Cys111 would reduce the binding affinity of (−)-γ-lactam and possibly the catalytic efficiency, which might explain the different catalytic specificities of the enantiomers of γ-lactam. Our results would facilitate the directed evolution and application of Mh33H4-5540 in antiviral drug synthesis.

2-Azabicyclo[2.2.1]hept-5-en-3-one (γ-lactam) has great potential as a synthetic intermediate for carbocyclic sugar amines, carbonucleosides, and carbocyclic dinucleotide analogues^1^. Particularly, the (−)-γ-lactam has been proved as an useful building block for the synthesis of the anti-HIV agents abacavir and carbovir^2^. Therefore, an efficient method is required for the preparation of the appropriate enantiomer of γ-lactam synthon. The (+)-γ-lactamase which catalyzes the enantioselective hydrolysis of (+)-γ-lactam but with no activity against the (−)-enantiomer (Fig. 1a) is thus industrially applied for the production of optically pure (−)-γ-lactam^3^,^4^.

Compared with chemical synthesis, enzymatic resolution of racemic γ-lactam with (+)-γ-lactamase has proven to be more efficient and environmentally friendly to give optically pure (−)-γ-lactam^5^. Over the past few decades, intensive efforts have been made to discover effective γ-lactamases by both screening and genome mining methods. To date, several (+)-γ-lactamases and (−)-γ-lactamases have been reported^6^,^7^,^8^,^9^,^10^,^11^,^12^,^13^,^14^,^15^. Although the three currently known (−)-γ-lactamases (1HKH_A from an *Aureobacterium* species, ACY56506.1 from *Microbacterium hydrocarbonoxydan*s and WP_014491492.1 from *Bradyrhizobium japonicum*) are similar in sequence to each other, the amino acid sequence are quite diverse among the known (+)-γ-lactamases due to the different subfamilies of (+)-γ-lactamases. All the (+)-γ-lactamases also show low sequence identities to (−)-γ-lactamases. A (−)-γ-lactamase which was isolated from an *Aureobacterium* species has been characterized and expressed in *Escherichia coli (E. coli*)^6^. The crystal structure was solved in complex with a covalently bound ligand BD1 ((3aR,7aS)-3a,4,7,7a-tetrahydro-benzo [1,3] dioxol-2-one), which originated from the host *E. coli* cells. Based on the complex structure, a mechanism of (−)-γ-lactam hydrolysis was suggested, which agreed with that of α/β hydrolase enzymes^16^. The crystal structure of a (+)-γ-lactamase from *Comamonas acidovorans* was also solved, which showed high sequence identity to formamidases and acetamidases^17^. However, although its crystal structure has been deposited in the Protein Data Bank (designated as PDB hereafter, accession code 2WKN), the reaction mechanism underlying the hydrolysis of (+)-γ-lactam remained elusive.

To date, all reported conventional γ-lactamases resemble formamidase or acetamidase homologs. Searches by using enzymes active on γ-lactam or amides as templates also facilitated the discovery of new enantiocomplementary γ-lactamase^15^. In our previous study, however, we identified an enzyme Mh33H4-5540 from *Microbacterium hydrocarbonoxydans* with a high (+)-γ-lactamase activity^18^, but very low sequence identity to the described (+)-γ-lactamases (5.7%, 4.8%, 7.2%, and 5.4% identities between its amino acid sequence and those of (+)-γ-lactamases from *Comamonas acidovorans, Bradyrhizobium japonicum, Aeropyrum pernix*, and *Sulfolobus solfataricus*, respectively)^18^. PSI-BLAST revealed that Mh33H4-5540 belongs to the isochorismatase-like hydrolase (IHL) superfamily^19^,^20^. Typically, Mh33H4-5540 showed 33% identities at the amino acid level with an isochorismatase (Pp1826) from *Pseudomonas putida* Kt2440 (PDB code 4H17) (Fig. 1c). In this view, Mh33H4-5540 (designated as MhIHL hereafter) constitutes a new type of (+)-γ-lactamase, in contrast to the described formamidase or acetamidase homologs.

The IHL superfamily members are α/β hydrolases, the first several characterized members of which all contained a conserved Cys residue (C111 in MhIHL in this study) which acts as a nucleophile at the active site, and therefore this family was formerly designated as cysteine hydrolases. However, further studies indicated that the cysteine residue is not fully conserved, since several members utilized other nucleophilic residues^21^. IHLs catalyze the hydrolysis of a variety of substrates^19^, out of which γ-lactam is never reported.

In the present study, we firstly conducted a hydrolysis assay of the racemic γ-lactam by MhIHL in an extended time range. Interestingly, the results showed that MhIHL actually showed both (+)- and (−)-γ-lactamase activity, but with apparently different specificities (Fig. 2a). To investigate the mechanisms underlying the hydrolysis of γ-lactams by MhIHL and its enantioselectivity, we solved the crystal structure of native MhIHL at 2.05 Å, and the structures of the inactive C111A variant in complex with (+)-γ-lactam and (−)-γ-lactam at 1.79 Å and 1.99 Å, respectively (Table 1). Structural and biochemical analysis confirmed the similar fold of MhIHL to other IHLs, and suggested a mechanism by which MhIHL facilitates γ-lactam hydrolysis. Further molecular dynamics (MD) simulation results of the enzyme-substrate complex structures clearly explained the different kinetic parameters and catalytic efficiencies of (+)- and (−)-γ-lactam. Taken together, our study represented the first structure of a (+)-γ-lactam/lactamase complex and also the first structure of a γ-lactam-bound IHL family member. Structural, biochemical, and MD simulation studies provided the basis of MhIHL-catalyzed hydrolysis of γ-lactam and the enantioselectivity of the enzyme. The study will also shed light on the further directed evolution and application of MhIHL and other IHL-family proteins in antiviral drug synthesis.

## Results

### MhIHL preferentially hydrolyzes (+)-γ-lactam versus (−)-γ-lactam

In our previous study^18^, by controlling the conversion under 50% (49.9%), we obtained the (−)-γ-lactam with enantiomeric excess (ee) (>99%) and the enantiomeric ratio (E value) (>200), which indicated the high enantioselectivity of MhIHL. We further extended the reaction time to investigate whether MhIHL exhibited absolute enantioselectivity towards (+)-γ-lactam. Interestingly, the results showed that MhIHL actually displayed hydrolysis towards both (+)- and (−)-γ-lactam, but with apparently different specificities (Fig. 2a and b). It appeared that MhIHL first hydrolyzed (+)-γ-lactam by high catalytic efficiency, and then it started to hydrolyze (−)-γ-lactam in a lower catalytic rate when the amount of the (+)-γ-lactam was negligible. The reaction finally reached a nearly 100% conversion (Fig. 2b), different from a typical enantioselective reaction with about 50% conversion^22^. Kinetic studies showed that the *Km* values of the (+)- and (−)-γ-lactam were 12.9 and 34.2 mM, respectively, indicating that MhIHL exhibited a better affinity towards (+)-γ-lactam than (−)-γ-lactam (Table 2). Furthermore, the *kcat*/*Km* value of (+)-γ-lactam (15.77 mM^−1^ s^−1^) is around 27 times that of (−)-γ-lactam (0.58 mM^−1^ s^−1^), suggesting the better specificity of MhIHL for (+)-γ-lactam. Together, MhIHL exhibited both hydrolytic activities towards (+)- and (−)-γ-lactam and a better specificity for (+)-γ-lactam.

### Overall Structure of native MhIHL

To better understand the different specificities of MhIHL for (+)- and (−)-γ-lactam, we firstly solved the high resolution structure of MhIHL (Table 1). Like other IHLs, MhIHL folds into a typical α/β domain with a six-stranded parallel β-sheet (β1-6) in the middle, flanked by three helices (α2-4), and a single long helix (α1) on both sides of the sheet, respectively (Fig. 2c). Relative to the sequence, the order of the strands in the sheet is 3-2-1-4-5-6. An additional 2-strand antiparallel β-sheet (residues 138–140 and 143–145, which corresponds to strands β1′ and β2′ in Fig. 2c) resides between β5 and α4, which is a unique feature shared by several IHL family proteins^20^. MhIHL crystallized in space group P3_1_21 with one polypeptide chain in the asymmetric unit. The PISA server^23^ predicts that MhIHL forms a dimer in solution, consistent with the gel filtration result of MhIHL (Supplementary Fig. S1). The dimer is formed mainly by the hydrophobic interactions between the 2-strand antiparallel β-sheet from one protomer and α4 from the other protomer. The total contact area of the monomer is 2526.1 Å^2^, and the decrease in this area upon dimerization is 488.6 Å^2^ in one protomer.

### Comparison with other IHL proteins

A search in the DALI server^24^ with the structure of MhIHL returns several results with Z scores ranging from 17.2 to 26.3, all of which are IHL superfamily members. SCOP^25^ divides this superfamily into six subgroups according to their catalyzed reactions which were pyrazinamidase/nicotinamidase E.C.3.5.1.19, nicotinamidase-related proteins, N-carbamoylsarcosine amidohydrolase (CSHase) E.C. 3.5.1.59, isochorismatase E.C. 3.3.2.1, ribonuclease, and a family of bacterial proteins of unknown function exemplified by the *Escherichia coli* ycac gene product. The overall “α-β-α sandwich” fold is highly conserved among MhIHL and other IHL proteins (Supplementary Fig. S2). In addition, a cis peptide bond is observed between residues A106 and Q107, which is a well conserved feature among IHL family members^19^. Out of the IHL proteins, MhIHL shows the highest structural similarity to a putative IHL protein with no defined function from *Oleispira antarctica* (PDB code 3LQY), with a root-mean-squared displacement (RMSD) of 1.3 Å between the two proteins. However, they are different in the loop regions between β1 and α1, β2 and β3, and β6 and α4 (Supplementary Fig. S2). PhzD protein from *Pseudomonas aeruginosa* (PDB code 1NF9) represents one of the best characterized IHL family proteins. PhzD, which belongs to the isochorismatase E.C. 3.3.2.1 subgroup, functions in the first step of phenazine synthesis, hydrolyzing 2-amino-2-deoxyisochorismate into trans-2,3-dihydro-3-hydroxyanthranilic acid and pyruvate. The RMSD between the structures of MhIHL and PhzD is 1.44 Å (Supplementary Fig. S2). PhzD lacks the 2-strand antiparallel β-sheet in MhIHL, but has two additional α helices in the C-terminal direction of the six-stranded β-sheet (Supplementary Fig. S2 and S3).

### Active Site

**Figure 1:**
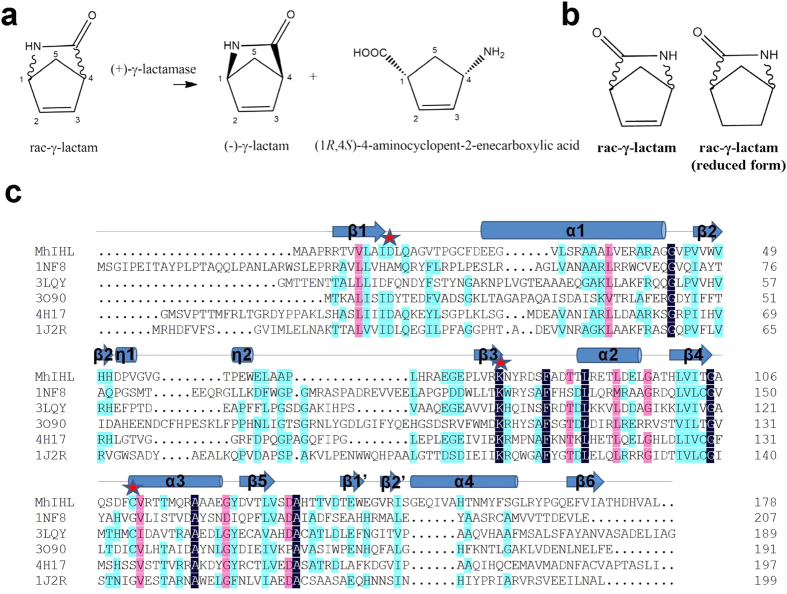
MhIHL is an isochorismatase-like superfamily member with (+)-γ-lactamase activity. (**a**) Kinetic resolution of (rac)-γ-lactam catalyzed by (+)-γ-lactamase. (**b**) Structures of racemic γ-lactam and its reduced forms. (C) Sequence alignment of IHL superfamily members from different species. The sequences of other IHL family members are marked with their respective PDB codes. The secondary structural elements of MhIHL are indicated above its amino acid sequence. The catalytic triad “D13-K78-C111” are marked with stars.

**Figure 2:**
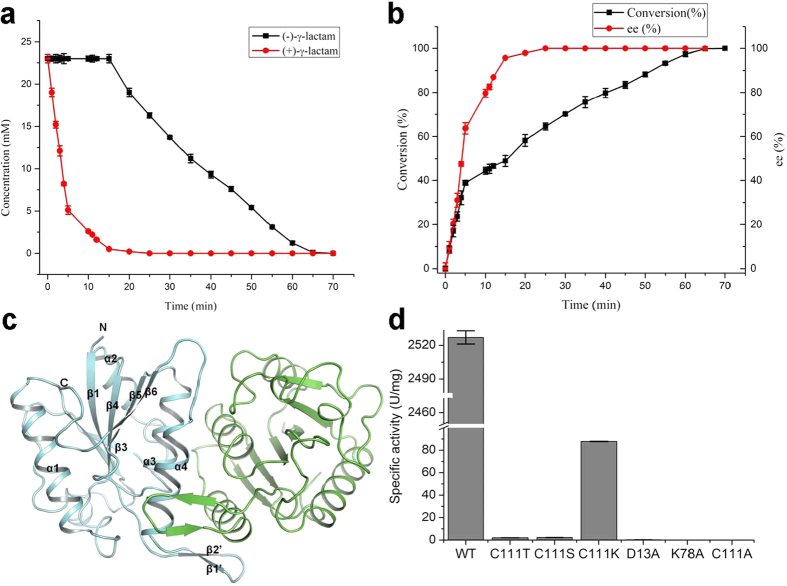
MhIHL preferentially hydrolyzes (+)-γ-lactam versus (−)-γ-lactam. (**a**) Progress curves for the hydrolysis of racemic γ-lactam. (**b**) Time course of enantioselective hydrolysis catalyzed by recombinant MhIHL. (**c**) MhIHL is shown in cartoon model with two protomers colored in cyan and green, respectively. The secondary structural elements of one protomer are indicated according to Fig. 1b. (**d**) The (+)-γ-lactamase activities of the wild type MhIHL and its mutants.

**Figure 3:**
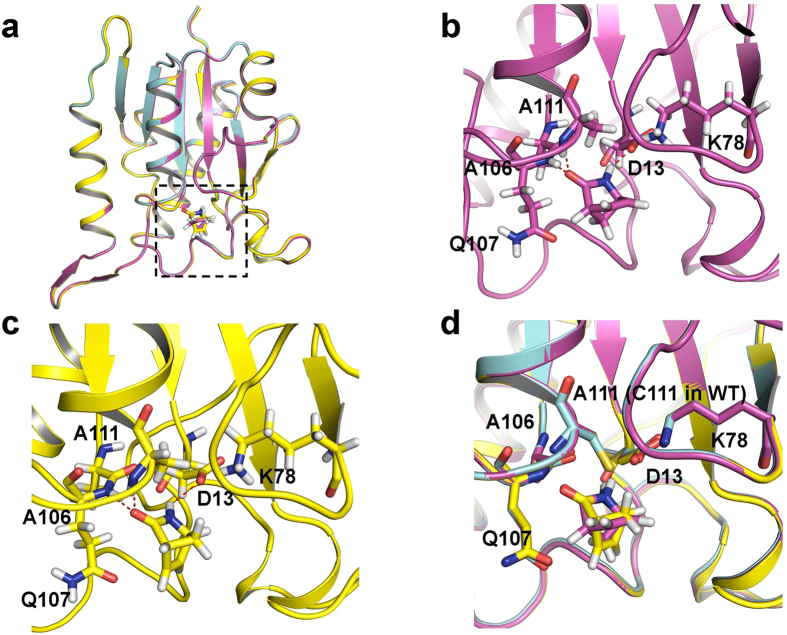
(+)- and (−)-γ-lactam bind the same site in MhIHL. (**a**) Overview of the superimpostion of the structures of apo-MhIHL, MhIHL-(+)-γ-lactam complex and MhIHL-(−)-γ-lactam complex. (**b**) Active site of the MhIHL-(+)-γ-lactam complex. The interacting residues are shown in sticks and the hydrogen bonds are shown as dashed lines. (**c**) Active site of the MhIHL-(−)-γ-lactam complex. The structure is shown as in Fig. 3b. (**d**) Superimposition of the active sites of apo-MhIHL, MhIHL-(+)-γ-lactam complex and MhIHL-(−)-γ-lactam complex.

Among the six subgroups of the IHL superfamily, three of the groups, namely the pyrazinamidase/nicotinamidase, ribonuclease and N-carbamoylsarcosine amidohydrolase proteins, share a D-K-C catalytic triad, while the others lack the nucleophilic cysteine (for example, D38-K122-G155 in PhzD). MhIHL also has this conserved triad (D13-K78-C111), and mutation of either residue to Ala caused sharp decrease or totally abolishment of the activity of this enzyme (Fig. 2d). Specifically, the D13A mutant showed 1/10 activity of wildtype enzyme (153.7 U/mg) and the K78A and C111A mutant enzymes almost showed no activities (Fig. 2d). This is consistent with the important roles the three residues played in the catalysis. Structures of the complex of MhIHL with either (+)-γ-lactam or (−)-γ-lactam were obtained by soaking the crystals of the C111A variant with the corresponding substrate, separately (Fig. 3). The active site of MhIHL is located at the C-termini of the six-stranded β-sheet, similar to other known structures with substrates (Fig. 3a). A clear electron density for one ligand was observed in the active site of each protomer (Supplementary Fig. S4) in both (+)- and (−)-γ-lactam-bound MhIHL structures. Surprisingly, (+)-γ-lactam and (−)-γ-lactam bind to nearly the same position in MhIHL (Fig. 3b–d). In this case, the backbone nitrogen atoms of these two residues formed the oxyanion hole which could stabilize the oxyanion transition state of the catalyzed reaction. In both structures, the carbonyl oxygen atom of the γ-lactam forms two hydrogen bonds with the main-chain nitrogen atoms of cis-Q107 and A111 (C111 in the native enzyme), respectively (Fig. 3b–d). In this case, the backbone nitrogen atoms of these two residues formed the oxyanion hole, which could stabilize the oxyanion transition state of the catalyzed reaction. The nitrogen atom of the ligand is hydrogen bonded with the side-chain of D13. The residues in the binding site are mostly conserved among isochorismatases (Fig. 1c). Although the γ-lactam binds to MhIHL through different mechanisms compared to other substrates in respective IHL proteins, the location of (+)-γ-lactam is typically the same as other substrates (Supplementary Fig. S3). PhzD contains two aforementioned additional α helices (α5: Thr83–Gly88 and α6: Leu90–Gly95) above the substrate cavity, together with the loops flanking them, serving as a “cover”, which could possibly slow down the release of the product from the cavity. This feature is conserved among the members whose active cavities are located totally in one protomer, and this “cover” could also be contributed by an adjacent protomer as seen in protozoan IHL proteins^19^. In MhIHL, however, this “cover” is lacking and could not be provided by the other protomer, either (Supplementary Fig. S3). We suggest that this open confirmation of the active cavity in MhIHL would facilitate both the binding of the substrate and the release of the product molecule, but simultaneously reduce the specificity of the substrate, consistent with the comparable enzymatic kinetic parameters of both enantiomers catalyzed by the MhIHL.

### Possible mechanism of γ-lactam hydrolysis

**Figure 4:**
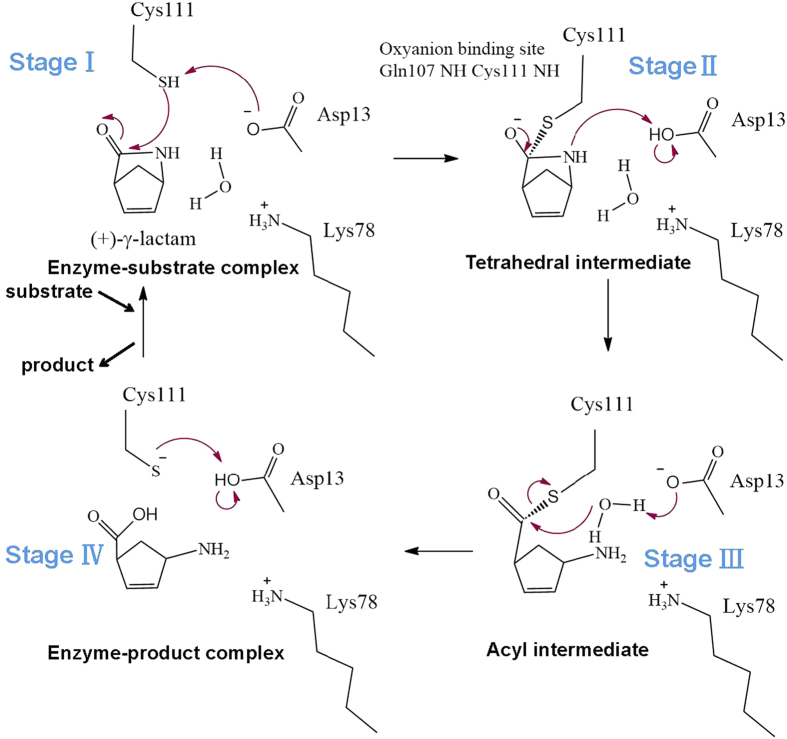
Proposed catalytic mechanism of MhIHL. Please see the main text for details.

A possible mechanism of MhIHL-catalyzed hydrolysis of (+)-γ-lactam or (−)-γ-lactam, is presented in Fig. 4, based on the structures of the C111A-(+)γ-lactam and C111A-(−)-γ-lactam complexes and the biochemical properties and mutational studies of the enzyme. In the mechanism, the carbonyl oxygen atom of the γ-lactam binds to the oxyanion hole formed by the main-chain nitrogen atoms of Q107 and C111 (Fig. 3b–d). In stage I of the modified mechanism, proton abstraction by D13 would generate a C111 thiolate facilitating nucleophilic attack at the carbonyl group, and the resulting tetrahedral intermediate is stabilized by the oxyanion hole. And then, proton donation from D13 to the ring nitrogen atom of the substrate would promote C-N bond cleavage and the tetrahedral intermediate collapses in stage II to give an acyl intermediate. In stage III, water activation by D13, whereby the D13 residue is protonated, generates a nucleophilic hydroxy group to attack the acyl intermediate to produce the product and a thiolate. In stage IV, the product is released and the thiolate accepts a proton from D13 to regenerate a thiol. In the whole mechanism, the K78 keeps its protonated form and does not directly participate in the catalysis process. This catalytic mechanism resembles the mechanism proposed for other α/β hydrolase enzymes such as *Acinetobacter baumanii* PncA^26^. In this mechanism, the thiol group of C111 acted as a nucleophilic group in the hydrolytic process. Mutation of C111 to other nucleophilic residues (C111T, C111S, and C111K) all caused sharp decrease of the activity (Fig. 2d), suggesting the indispensible role of thiol group in the active site. The slightly higher activity of C111K than that of C111T/S suggested a possible weak nucleophilic activity of K111 in the active site.

### Molecular dynamic (MD) simulations reveal the basis of the enantioselectivity of MhIHL

**Figure 5:**
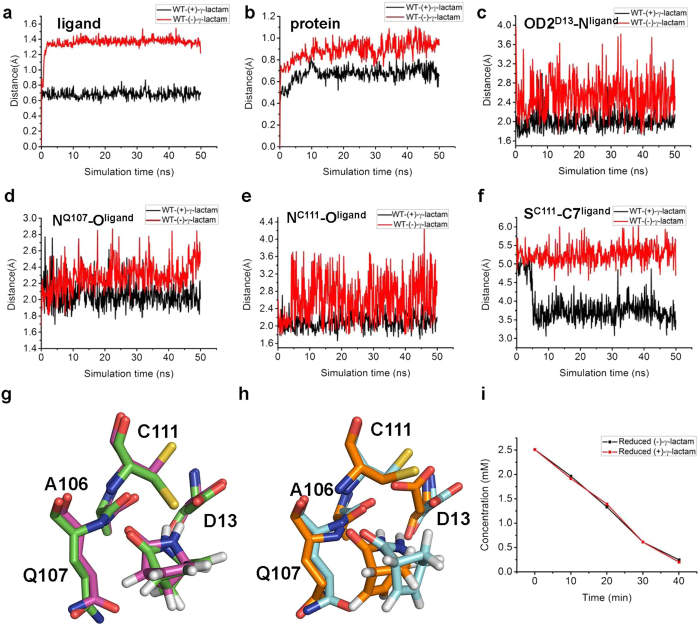
MD simulation results for the (+)−/(−)-γ-lactam-MhIHL complexes. The RMSDs of the ligands (**a**) and MhIHL (**b**) as a function of time with respect to the starting structure. (**c**–**e**) Investigation of the hydrogen bonds formed between D13, Q107, and C111 of MhIHL and the ligands. The distances between OD2 of D13 and N of the ligand (**c**), N of Q107 and O of the ligand (**d**), and N of C111 and O of the ligand (**e**), corresponding to HB1-3 in Fig. 3d, were shown as a function of time. (**f**) The distances between the nucleophilic sulfur atom of C111 and carbonyl C of the ligand in two complexes were shown as a function of time. (**g**) Active site conformation before (colored in magenta) and after (colored in green) 50 ns simulation of the (+)-γ-lactam-MhIHL complex. The four residues in the active site and (+)-γ-lactam are shown. (**h**) Active site conformation before (colored in cyan) and after (colored in orange) 50 ns simulation of the (−)-γ-lactam-MhIHL complex. The four residues in the active site and (−)-γ-lactam are shown. (**i**) Progress curves for the hydrolysis of racemic double-bond reduced γ-lactam by MhIHL. The reaction was carried out under the following conditions: 3 μg of purified MhIHL enzyme to 1000 μL of the 5 mM substrate solution (50 mM Tris-HCl, pH 7.5), 25 °C for 40 min.

As both enantiomers of γ-lactam bound to almost the same sites in MhIHL and formed similar interactions with the enzyme, we performed MD simulations to explore the molecular mechanism underlying the different catalytic specificities of the (+)-γ-lactam or (−)-γ-lactam of MhIHL. First, we constructed the structural models of the complex structures of the WT enzyme with (+)- or (−)-γ-lactam separately, based on the complex structures of C111A mutant with (+)- or (−)-γ-lactam using the Protein Local Optimization Program (PLOP). After 50 ns simulation, both complex systems of WT-(+)-γ-lactam and WT-(−)-γ-lactam reached equilibrium, while the average RMSD values of ligands and deviations of proteins were both larger in the WT-(−)-γ-lactam complex than in the WT-(+)-γ-lactam complex (Fig. 5a and b). During the simulation, (+)-γ-lactam could be well accommodated in the binding site of the WT enzyme and stayed stably in the equilibrium position (Fig. 5a and g). However, the (−)-γ-lactam deviated from the starting position with a larger RMSD after the 50 ns simulation (Fig. 5a and h). In addition, the three hydrogen bond interactions (Fig. 3b and C), which are essential for the binding of the γ-lactam to the enzyme, were also more disturbed in WT-(−)-γ-lactam system than in the WT-(+)-γ-lactam complex (Fig. 5c–e). The molecular mechanics Poisson-Boltzmann/surface area (MM-PB/SA) calculations also suggested the (+)-γ-lactam exhibited a better binding energy than (−)-γ-lactam, probably due to the more stable hydrogen bond network (Table 3). As the nucleophilic attack of the carbonyl group of the γ-lactam by the thiol group of C111 was suggested as an essential step in the reaction, the distance between the sulfur atom of C111 and the carbon atom of the γ-lactam was also investigated in both structures. In the WT-(+)-γ-lactam complex, the distance was stabilized at around 3.5 Å, a reasonable distance for the nucleophilic reaction to happen, in contrast to the distance greater than 5 Å in the WT-(−)-γ-lactam complex (Fig. 5f). Based on this result, we speculated that the bulkier sp3 bridged carbon atom C1 of (−)-γ-lactam might generate steric clash with the binding-site residues, especially the sidechain of C111, which resulted in the unstable binding of (−)-γ-lactam and its low catalytic efficiency, which might elucidate the enantioselectivity of MhIHL (Fig. 5g and h). To test this hypothesis, we also carried out MD simulations for the complex structures of C111A mutant with (+)- or (−)-γ-lactam (Supplementary Fig. S5). The results showed that the average RMSD values of ligands and deviations of proteins were both similar between the two complexes (Supplementary Fig. S5). Moreover, the differences of the occupancies of the three above mentioned hydrogen bonds between (+)- and (−)-γ-lactam-bound structures were also decreased in the C111A complexes, compared to the WT complexes (Supplementary Fig. S5), indicating that both (+)-γ-lactam and (−)-γ-lactam could be well accommodated in the binding pocket of the C111A enzyme (Supplementary Fig. S5). The gap of the calculated binding energies were also reduced between the systems of C111A mutant with (+)- and (−)-γ-lactam (Table 3). Moreover, since the saturated C1 atom and unsaturated C3 and C4 atoms constitute the major determinants of enantioselectity, we also tested the hydrolysis of hydrogenation-reduced form of γ-lactam enantiomers (Fig. 1b) by MhIHL (Fig. 5i). The results showed that MhIHL hydrolyzed both reduced enantiomers at similar rates (Fig. 5i). This suggested that both enantiomers of the reduced forms of γ-lactam might cause similar steric clashes with the enzyme, further supporting our proposal that the saturated C1 atom and unsaturated C3 and C4 atoms constitute the major enantioselectity determinants of γ-lactam by MhIHL.

## Discussion

Specific hydrolysis of (+)-γ-lactam out of racemic γ-lactam to leave optically pure (−)-γ-lactam is of great importance, since (−)-γ-lactam is the key building block of antiviral drugs such as carbovir and abacavir. To date, several (+)-γ-lactamases have been identified, all of which resemble formamidases and acetamidases, but no structure has been described for the complex between (+)-γ-lactam and a (+)-γ-lactamase. The IHLs superfamily is a well-characterized hydrolase family, which is best known in pathogenic organisms^19^. Recently, several structures of isochorismatases or putative isochorismatases have been elucidated. However, most of the isochorismatase homologs were poorly characterized biochemically and enzymatically. Moreover, no γ-lactamase activity has been reported for the members in this family. This study is the first report of the structures and mechanisms of an IHL family member with γ-lactamase activity.

Although the quaternary structures observed for members of this family are quite diverse, their tertiary α/β hydrolase fold is highly homologous, and key features of the active site are also conserved. First, most of the family numbers contain a D-K-C catalytic triad (D13-K78-C111 in MhIHL), out of which the thiol group of the cysteine residue acts as a nucleophile. Second, all structures determined to date from this superfamily exhibit a rare nonproline cis-peptide bond (A106-Q107 in MhIHL) in the active site, which has been proposed to help position the substrate-binding residues appropriately^27^. Inspired by this, we propose that members in the IHL family harboring these two features should also have the γ-lactamase activity. Consistently, another three IHL family proteins (designated isochorismatases A, B, and C, respectively) all displayed (+)-γ-lactamase activities (Supplementary Table 1), although the activities of these enzymes are lower than that of MhIHL. We also tested the activity of an isochorismatases-like protein Yecd (isochorismatase D) which lacks the suggested nucleophilic cysteine (D26-K112-G145 in Yecd). Yecd displayed no γ-lactamase activity, further supporting the indispensible role of a nucleophilic residue, such as cystein, serine, threonine, *et al*. (Supplementary Table 1).

Interestingly, Hamano *et al*. reported that the enzyme SttH from *Streptomyces albulus* could catalyze the hydrolysis of the amide bond of streptothricins (STs), which are broad-spectrum antibiotics. Streptothricins (STs), produced by *Streptomyces* strains, consist of a carbamoylated D-gulosamine to which the β-lysine homopolymer (1 to 7 residues) and the amide form of the streptolidine (streptolidine lactam) are attached^28^. The authors found that upon hydrolysis of the amide bond of streptolidine lactam, the toxicity of ST-D (possessing three-β-lysine moiety) was altered from broad-spectrum to bacteria-specific. They showed that hydrolyzed form of ST-D (ST-D-acid) exhibits potent antibacterial activity but its toxicity against eukaryotic cells is reduced. SttH is also a 276-amino-acid IHL family member, with 28% identity to MhIHL among 178 residues. A catalytic D-K-C triad (D70-K143-C176) also exists in SttH, and it will be interesting to investigate whether SttH catalyzes the hydrolysis of the amide bond of streptolidine lactam through the same mechanism as MhIHL, although the binding mechanism must be different due to the more complex structure of ST-D.

MhIHL exhibited different hydrolysis activities towards (+)- and (−)-γ-lactam, with a better specificity for (+)-γ-lactam. Therefore, in the beginning of the hydrolysis reaction of racemic γ-lactam, (+)-γ-lactam might act both as a substrate of MhIHL, and also an “inhibitor” for (−)-γ-lactam. That explains why it seems that MhIHL first hydrolyzed the (+)-γ-lactam, and then the (−)-γ-lactam (Fig. 2a). To investigate the mechanism underlying the different specificities towards (+)-γ-lactam and (−)-γ-lactam, we solved the structures of apo-form MhIHL, and both substrate-bound complex with the C111A mutant enzyme, and performed MD simulation studies. The structures revealed that both γ-lactam enantiomers bind to almost the same position and their nitrogen and oxygen atoms are stabilized by similar hydrogen bonds. However, MD simulation results suggested that the bulkier sp3 bridged carbon atom C1 of (−)-γ-lactam might generate steric clash with the binding-site residues of MhIHL, especially the side chain of C111, which would decrease the binding specificity of the (−)-γ-lactam in MhIHL and possibly the catalytic efficiency as well. In the (+)-γ-lactam-bound complex structure, however, the thiol group of C111 is facing the unsaturated carbon atoms C3 and C4, leading to less steric clash. Importantly, although the differences of the occupancies of the three hydrogen bonds between (+)- and (−)-γ-lactam-bound structures were decreased in the C111A complexes, (−)-γ-lactam still could not be accommodated well in the binding cavity (Supplementary Fig. S5), suggesting that other residues in the binding site could also contribute to the enantioselectivity of MhIHL. In addition, the similar hydrolysis rate of MhIHL towards the reduced forms of (+)- and (−)-γ-lactam (Fig. 5i) further supported that the saturated C1 atom and the unsaturated C3 and C4 atoms might be the determinants of the enantioselectivity towards racemic γ-lactam of MhIHL.

In all, we have presented three structures of MhIHL and its complex with both (+) and (−)-γ-lactam substrate and conducted biochemical and mutational studies into the catalytic mechanism and enantioselectivity of MhIHL. MhIHL from the isochorismatase-like superfamily, represents a novel class of (+)-γ-lactamases, thus further expanding the (+)-γ-lactamase superfamily. MhIHL displayed different hydrolysis activities towards (+)- and (−)-γ-lactam, and our structural, biochemical, and MD simulation studies demonstrated the underlying mechanisms for the enantioselectivity of MhIHL. Due to the wide applications of (−)-γ-lactam in antiviral drugs, this study provided much needed insight into the catalytic mechanism of a new type of (+)-γ-lactamase and would shed light on the molecular manipulation of (+)-γ-lactamases for higher efficiency and enantioselectivity.

## Materials and Methods

### Construction of expression plasmids and the mutants

*M. hydrocarbonoxydans, Paenibacillus dendritiformis C454, Paenibacillus polymyxa* SQR-21, *Microbacterium testaceum* StLB037, and *Escherichia coli* W3110 were obtained from the China General Microbiological Culture Collection Center, and grown in Luria-Bertani (LB) medium at 37 °C. The gene encoding MhIHL (GenBank accession number AKS37009.1) from *M. hydrocarbonoxydans*, isochorismatase A from *P. dendritiformis* C454 (GenBank accession number CP003422.2), isochorismatase B from *P. polymyxa* SQR-21(GenBank accession number CP006872.1), isochorismatase C from *M. testaceum* StLB037 (GenBank accession number AP012052.1), and isochorismatase D from *E. coli* W3110 (GenBank accession number CP013253.1) were amplified by PCR from the corresponding genomic DNA. The amplified genes were purified using a Gel/PCR Extraction kit (QIAGEN, Germany), digested by NdeI and XhoI (New England Biolabs, Beverly, MA), and inserted into pET22b(+) (Novagen, Madison, WI), resulting in plasmids encoding a His_6_ tag fusion at the C terminal of the recombinant proteins. Mutants were generated by site-directed mutagenesis with a QuikChange Site-Directed Mutagenesis Kit (Agilent Technologies, Santa Clara, CA, USA). The expression plasmids for wild-type (WT) and the mutants were introduced into *E. coli* DH5α and *E. coli* DMT respectively and verified by DNA sequencing.

### Expression and purification of proteins

*E. coli* Rosetta (DE3) cells harboring recombinant plasmids were grown in LB medium containing ampicillin (50 μg/mL). The cells were induced with isopropyl β-D-thiogalactopyranoside (IPTG) at a final concentration of 0.1 mM when the absorbance reached 0.8–1.0 at 600 nm. The incubation was then continued at 30 °C for another 5 h. The cells were harvested by centrifugation at 3,500 × g for 10 min, resuspended in 50 mM Tris-HCl (pH 7.8), and disrupted by sonication. The cell debris was removed by centrifugation at 12,000 × g for 50 min. The cell-free extract obtained was applied onto a nickel nitrilotriacetic acid (Ni-NTA) agarose (GE Healthcare) column equilibrated with 20 mM imidazole in 50 mM Tris-HCl buffer (pH 8.0). Next, the enzyme was further purified on a Superdex 200 column (GE Healthcare) with a 50 mM Tris-HCl buffer (pH 7.8) containing 300 mM NaCl and 2 mM β-mercaptoethanol using AKTA FPLC system (GE Healthcare). The protein concentration was determined spectrophotometrically at 280 nm using the Thermo NanoDrop 2000 and further calibrated according to the theoretical extinction coefficient based on the amino acid sequences. The MhIHL mutants and other isochorismatases were expressed and purified in a manner identical to that used for the WT MhIHL enzyme.

### Measurement of enzymatic activity and kinetic constants

The catalytic activities of MhIHL and mutants were examined by the addition of 0.2 μg pure MhIHL to 1000 μL of the 46 mM substrate solution (50 mM Tris-HCl, pH 7.5). The catalytic activities of isochorismatase A, B, C, and D were detected by the addition of 80 μg pure enzyme to 1000 μL of the 46 mM substrate solution (50 mM Tris-HCl, pH 7.5). The reactions were incubated at 25 °C for 15 min and then extracted with 1000 μL ethyl acetate. The (+)-γ-lactamase activity was detected as described previously^10^. One unit (U) of enzyme activity was defined as the amount of enzyme that catalyzed the conversion of one nanomole of substrate per minute. Measurements of kinetic parameters for bioresolution reactions were performed at 25 °C. The hydrolyzing reactions were performed in 50 mM Tris-HCl (pH 7.5) with (−)-γ-lactam or (+)-γ-lactam as the substrates. The concentration of the γ-lactam was varied from 5 to 200 mM. All the experiments were conducted with three replicates. *Km* and *V*_max_ values were calculated by nonlinear fitting. Conversion and enantiomeric excess (ee) were calculated as previously described^18^.

### Crystallization

All the crystals were obtained at 20 °C using the sitting drop vapor diffusion method by mixing 1 μL of protein solution (15 mg/mL) with an equal volume of reservoir solution containing 0.2 M sodium malonate (pH 6.0) and 20% (w/v) polyethylene glycol 3,350. Crystals grew to a full size in two or three days. Crystals were cryoprotected in the reservoir solutions supplemented with 25% (w/v) glycerol, flash cooled in the liquid nitrogen, and stored at cryogenic temperatures for data collection. Both co-crystallization and soaking methods were conducted to obtain the enzyme-substrate structure. The final crystals of MhIHL–(−)-γ-lactam complex were obtained by soaking the crystal of the substrate-free C111A mutant enzyme in a cryoprotectant containing 250 mM (−)-γ-lactam for 1.5 h. The final MhIHL–(+)-γ-lactam complex crystals were obtained by soaking the crystal of substrate-free C111A mutant enzyme in cryoprotectant containing 500 mM (+)-γ-lactam for 10 min.

### Data collection, and structure determination

Diffraction data were collected at the Shanghai Synchrotron Radiation Facility (SSRF), integrated and scaled using HKL2000^29^. The initial phase calculation, phase improvement, and automated model building were performed using the molecular replacement (MR) method with PHASER in the CCP4 program suite^30^ and PHENIX suite^31^. The structure of an isochorismatase (Pp1826) from *Pseudomonas putida* Kt2440 (PDB code 4H17) was used as the search model. The final model rebuilding was performed using COOT^32^ and the protein structure was refined with PHENIX^33^ against the data using stereochemistry information. For the complex of C111A mutant with either (+)-γ-lactam or (−)-γ-lactam, the molecular replacement method was also used with the structure of the native enzyme as the search model. The subsequent model building and refinement was performed as mentioned for the native enzyme. Data-collection and refinement statistics are presented in Table 1. All structural model figures were created with PyMOL^34^.

### Molecular dynamics (MD) simulation

To further explore the molecular mechanism of enantioselectivity of the enzyme, MD simulations were carried out for the complex structures of the WT/C111A enzyme with (+)-γ-lactam and (−)-γ-lactam respectively. The initial structures of the two WT complexes were modeled based on the C111A mutant-(+)/(−)-γ-lactam crystal structures using Protein Local Optimization Program (PLOP)^35^,^36^,^37^. The MD simulations were carried out using AMBER10.0 suite^38^. The AMBER99SB force filed^39^ was applied for protein and the general Amber force field (GAFF)^40^ for the ligands. ANTECHARMBER was used for calculation of the AM1-BCC charges of ligands. All system setups were performed in TLEAP module. A 12 Å pad of TIP3P waters was added to solvate to each system. Neutralizing counter ions were added to each system. Simulations were carried out using the SANDER module. Each system was minimized using 5000 steps of steepest descent algorithm with gradually reduced restraint. Following minimization, the system was gradually heated to 300 K in the canonical NPT ensemble using a Berendsen thermostat^41^, with a 50 kcal/mol/Å^2^ harmonic position restraint applied to all heavy atoms of the protein. The restraints were gradually removed and the production run was performed in MTK-NPT (1 bar, 300 K) ensemble for 50 ns (WT enzyme) and 10 ns (C111A enzyme), respectively. The SHAKE algorithm^42^ was applied to constrain all bonds involving hydrogen atoms with a time step of 2 fs.

## Additional Information

**Accession codes**: The coordinates and structure factors of the apo-form, (＋)-γ -lactam bound, and (ý)-γ -lactam bound of MhIHL have been deposited in the Protein Data Bank under accession codes 5HA8, 5HWH and 5HWG.

**How to cite this article:** Gao, S. *et al*. Structural insights into the γ-lactamase activity and substrate enantioselectivity of an isochorismatase-like hydrolase from *Microbacterium hydrocarbonoxydans. Sci. Rep.* **7**, 44542; doi: 10.1038/srep44542 (2017).

**Publisher's note:** Springer Nature remains neutral with regard to jurisdictional claims in published maps and institutional affiliations.

## Supplementary Material

Supplementary Information

## Figures and Tables

**Table 1:** Statistics of data collection and structure refinement.

Data collection	MhIHL	MhIHL-(+)-γ-lactam	MhIHL-(−)-γ-lactam
Space Group	P3_1_21	P3_1_21	P3_1_21
Unit Cell (Å)	69.454, 69.454, 88.052	69.427, 69.427, 87.159	69.291, 69.291, 86.617
Wavelength (Å)	0.979	0.979	0.979
Resolution (Å)	2.05 (2.13–2.05)	1.79 (1.885–1.791)	1.99 (2.06–1.99)
Rsym (%)	11.5 (65.6)	5.9 (15.2)	9.0 (54.4)
I/sigma	20.17 (3.77)	32.74 (17.74)	22.3 (4.9)
Completeness (%)	99.77 (97.74)	98.19 (99.91)	98.5 (92.5)
Redundancy	13.1 (12.3)	11.1 (12.0)	9.4 (8.8)
Wilson B factor (Å^2^)	42.66	17.43	21.97
Refinement			
R factor	0.1709	0.1921	0.2014
Rfree	0.1941	0.2199	0.2390
No. atoms	1339 protein atoms + 139 waters	1338 protein atoms + 8 ligand atoms + 230 waters	1338 protein atoms + 8 ligand atoms + 107 waters
B factors			
Overall	43.1	23.0	24.9
Macromolecules	42.4	21.1	24.3
ligand		22.5	22.0
solvent	49.6	34.2	32.7
RMSD bond lengths	0.004	0.004	0.004
RMSD bond angles	0.89	0.85	0.8
Ramachandran plot statistics (%)			
In preferred regions	98	98	98
In allowed regions	2	2	2
Ourliers	0	0	0
PDB code	5HA8	5HWH	5HWG

Values in parentheses are for the highest resolution shell. *R*_sym_ = Σ_h_Σ_i_|*I*_*h,i*_ − *I*_*h*_|/Σ_h_Σ_i_*I*_*h,i*_, where *I*_*h*_ is the mean intensity of the *i* observations of symmetry related reflections of *h. R* = Σ|*F*_*obs*_ − *F*_*calc*_|/Σ*F*_*obs*_, where *F*_*calc*_ is the calculated protein structure factor from the atomic model (R_free_ was calculated with 5% of the reflections).

**Table 2:** Kinetic constants of the MhIHL towards (+)-γ-lactam and (−)-γ-lactam.

Parameters	(+)-γ-lactam			(−)-γ-lactam		
	*Km* (mM)	*kcat* (s^−1^)	*kcat*/*Km* (mM^−1^ s^−1^)	*Km* (mM)	*kcat* (s^−1^)	*kcat*/*Km* (mM^−1^ s^−1^)
MhIHL	12.9	203.433	15.77	34.2	19.836	0.58

**Table 3:** Calculated binding energy (Unit: kcal/mol) by molecular mechanics Poisson-Boltzmann/surface area (MM-PB/SA).

	ΔG_binding_	ΔE_elec_	ΔE_vdW_	ΔG_polar_	ΔG_nonpolar_
(+)-γ-lactam-MhIHL	−18.67 ± 2.09	−21.21 ± 2.18	−16.86 ± 1.87	22.06 ± 2.08	−2.66 ± 0.02
(−)-γ-lactam-MhIHL	−15.94 ± 1.96	−18.22 ± 3.12	−15.36 ± 1.78	20.21 ± 1.81	−2.60 ± 0.03
(+)-γ-lactam-C111A	−17.51 ± 2.69	−21.13 ± 3.87	−16.18 ± 2.00	22.49 ± 2.94	−2.69 ± 0.02
(−)-γ-lactam-C111A	−16.86 ± 3.25	−19.82 ± 4.20	−15.67 ± 1.52	21.27 ± 4.11	−2.64 ± 0.03
