# Supplementary material for: Structural insights into the γ-lactamase activity and substrate enantioselectivity of an isochorismatase-like hydrolase from Microbacterium hydrocarbonoxydans
Source: Sci Rep. 2017 Mar 15;7:44542. doi: 10.1038/srep44542 (PMC5353710; doi:10.1038/srep44542)
Supplement: Supplementary Information [file srep44542-s1.pdf]

Structural insights into the γ-lactamase activity and substrate enantioselectivity of an isochorismatase-like hydrolase from *Microbacterium hydrocarbonoxydans*

Shuaihua Gao[1], Yu Zhou[2], Weiwei Zhang[1], Wenhe Wang[1], You Yu[3], Yajuan Mu[1], Hao Wang[1], Xinqi Gong[4], Guojun Zheng[1*] and Yue Feng[1*]

1 Beijing Key Lab of Bioprocess, the State Key Laboratory of Chemical Resources Engineering, College of Life Science and Technology, Beijing University of Chemical Technology, Beijing 100029, PR China

2 National Institute of Biological Sciences, Beijing, No. 7 Science Park Road, Zhongguancun Life Science Park, Beijing 102206, PR China

3 Key Laboratory for Protein Sciences of Ministry of Education, Tsinghua-Peking Center for Life Sciences, School of Life Sciences, Tsinghua University, 100084, Beijing, PR China.

4 Institute for Mathematical Sciences, Renmin University of China, Beijing 100872, PR China

To whom correspondence should be addressed: Yue Feng, Beijing University of Chemical Technology, Beijing, 100029, China, Tel. /fax: +8601064437507, E-mail: fengyue@mail.buct.edu.cn. Guojun Zheng, Beijing University of Chemical Technology, Beijing, 100029, China, Tel. /fax: + 86 01064437507, E-mail: zhenggj@mail.buct.edu.cn;

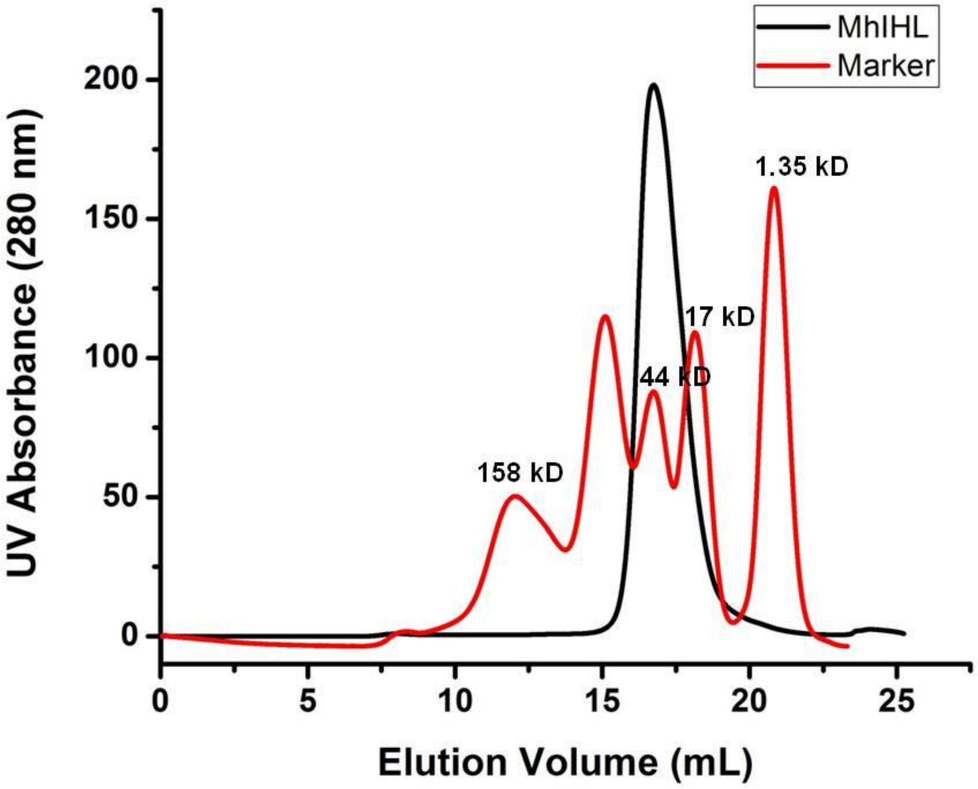

Supplementary Fig. S1 MhIHL forms a stable homodimer in solution.

The gel filtration profiles of MhIHL and the molecular markers on Superdex-200 column are shown. The sizes of the molecular markers are marked on top of the peaks.

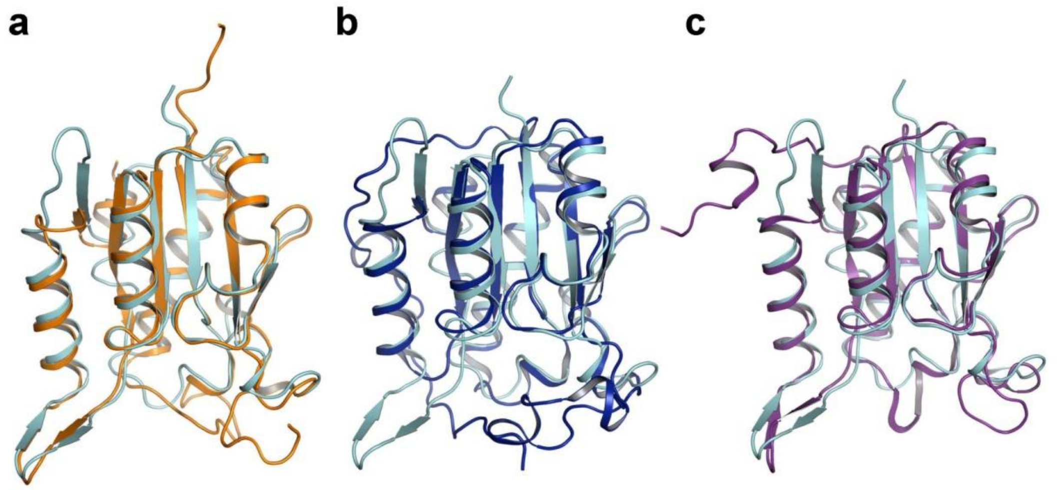

Supplementary Fig. S2 Comparison of MhIHL with other IHL family proteins. (A) Superimposition of MhIHL (cyan) and OaIHL from *Oleispira antarctica* (orange) (PDB: 3LQY). (B) Superimposition of MhIHL and PhzD from *Pseudomonas aeruginosa* (blue) (PDB: 1NF9). (C) Superimposition of MhIHL and a putative isochorismatse from Pseudomonas putida KT2440 (magenta) (PDB: 4H17).

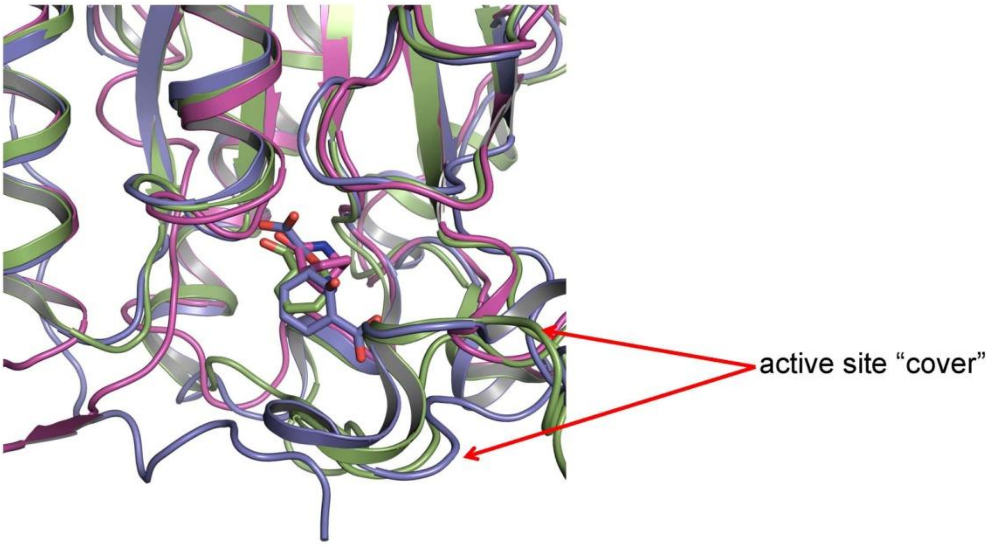

Supplementary Fig. S3 Comparison of the active pockets among MhIHL and other IHL family proteins. Superimposition of the complex between MhIHL-(+)-γ-lactam (light magenta), PhzD-isochorismate from *Pseudomonas aeruginosa* (slate) (PDB: 1NF8) and nicotinamidase-nicotinamide complex (splitpea) (PDB: 3O94). The regions corresponding to the active site "covers" in PhzD-isochorismate and nicotinamidase-nicotinamide are indicated.

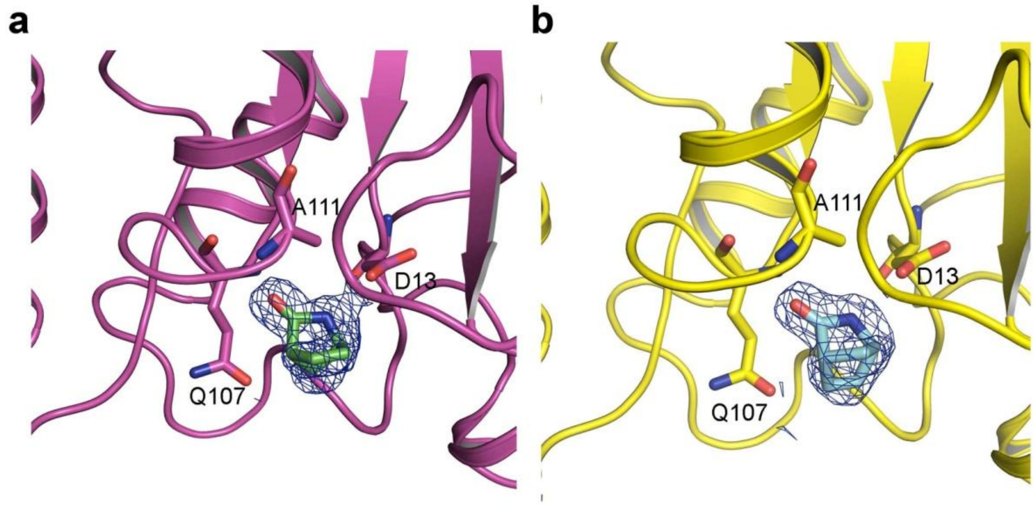

Supplementary Fig. S4 The electron densities of the ligands. (A) The active site of the MhIHL-(+)-γ-lactam complex. (B) The active site of the MhIHL-(-)-γ-lactam complex. The 2Fo-Fc electron density maps (1.0 σ) of the ligands are shown as blue mesh.

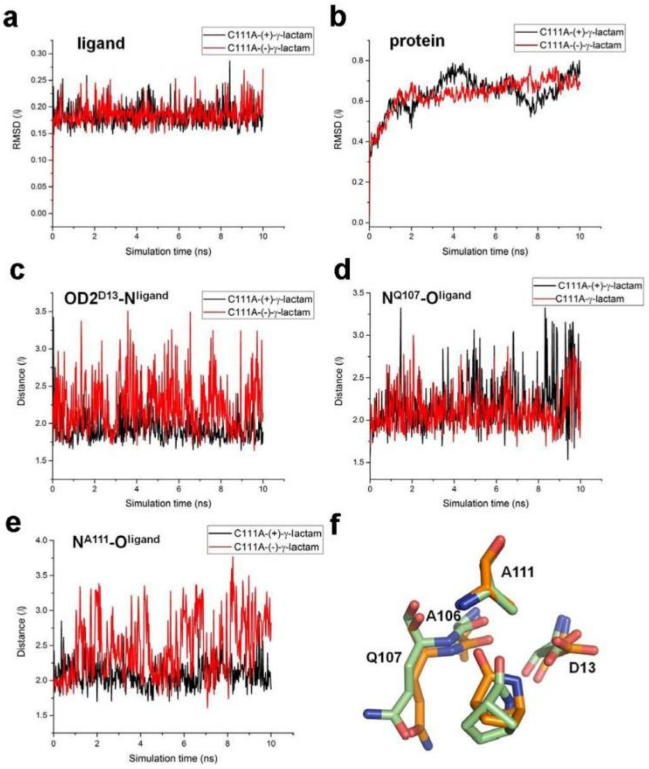

Supplementary Fig. S5 MD simulation results for the (+)/(-)-γ-lactam-C111A complexes. The RMSDs of the ligands (A) and C111A mutant (B) as a function of time with respect to the starting structure. (C-E) Investigation of the hydrogen bonds formed between D13, Q107, and C111 of C111A and the ligands. The distances between OD2 of D13 and N of the ligand (C), N of Q107 and O of the ligand (D), and N of C111 and O of the ligand (E), corresponding to HB1-3 in Fig. 3D, were shown as a function of time. (F) The location of (+)/(-)-γ-lactam in the active sites of C111A enzyme after 10 ns simulation. The ligands and active site residues are shown as sticks. The complex structures of (+)-γ-lactam-C111A and (-)-γ-lactam-C111A after 10 ns simulation are colored in orange and green, respectively.

Supplementary Table 1. (+)-γ-lactamase activity detection of MhIHL and four selected isochorismatases

Organism	Name	Accession Number	Catalytic triad	(+)-γ-lactamase activity (U/mg)
M.	MhIHL	AKS37009.1	D13-K78-C111	2527.0±5.9

hydrocarbonoxydans				
P. dendritiformis C454	Isochorismatase A	CP003422.2	D9-K86-C119	18.5±8.5
P. polymyxa SQR-21	Isochorismatase B	CP006872.1	D10-K83-C116	150.6±50.1
M. testaceum StLB037	Isochorismatase C	AP012052.1	D9-K84-C118	126.7±26.1
E. coli W3110	Isochorismatase D	CP013253.1	D26-K113-G145	0
